# Supplementary material for: Loss-of-function mutation in PRMT9 causes abnormal synapse development by dysregulation of RNA alternative splicing
Source: Nat Commun. 2024 Apr 1;15:2809. doi: 10.1038/s41467-024-47107-9 (PMC10984984; doi:10.1038/s41467-024-47107-9)
Supplement: Supplementary file 1 — Supplementary Information [file 41467_2024_47107_MOESM1_ESM.pdf]

Supplementary Information

Supplementary Fig. 1

a

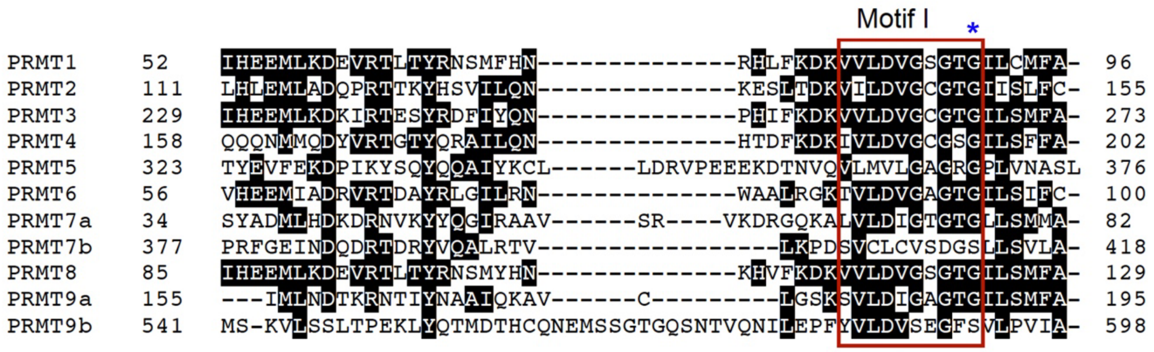

b

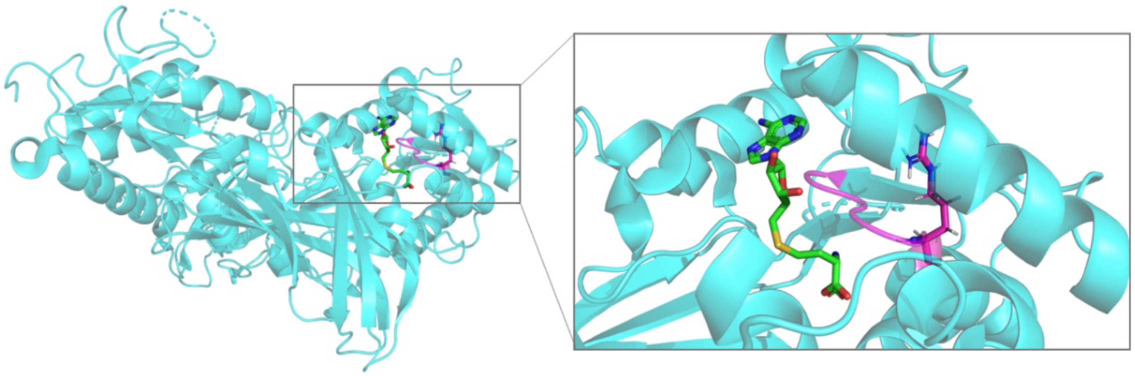

Supplementary Fig. 1: Amino-acid sequence alignment and structural analysis of PRMT9.

a. The amino-acid sequences from Motif I of the catalytic domain of PRMTs are compared using ClustalW. Motif I located at the N-terminus of the catalytic domain is boxed in red. Numbers indicate amino acid positions of the individual PRMTs. Human PRMT sequences used for alignment included PRMT1: NP_001527.3; PRMT2: NP_996845.1; PRMT3: NP_005779.1; PRMT4: NP_954592.1; PRMT5: NP_006100.2; PRMT6: NP_060607.2; PRMT7: NP_061896.1; PRMT8: NP_062828.3; and PRMT9: NP_612373.2. The blue asterisk indicates the conserved Glycine that is mutated in PRMT9 in patients with ID.

b. SAM bound PRMT7 structure (PDB ID: 4C4A) was aligned with PRMT9 structure (PDB ID: 6PDM, aqua in cartoon representation) to position SAM (green in stick representation) in PRMT9 SAM-binding pocket module N (RMSD over 2341 atoms is 3.229). Gly189 resides in the conserved Motif I (VLD/VGxGxG) (magenta). Mutation of this residue to arginine (represented in sticks) may distort the conserved Motif I loop or destabilize the overall fold of SAM-binding pocket. The G189R mutation in apo PRMT9 was modeled in PyMOL.

Supplementary Fig. 2

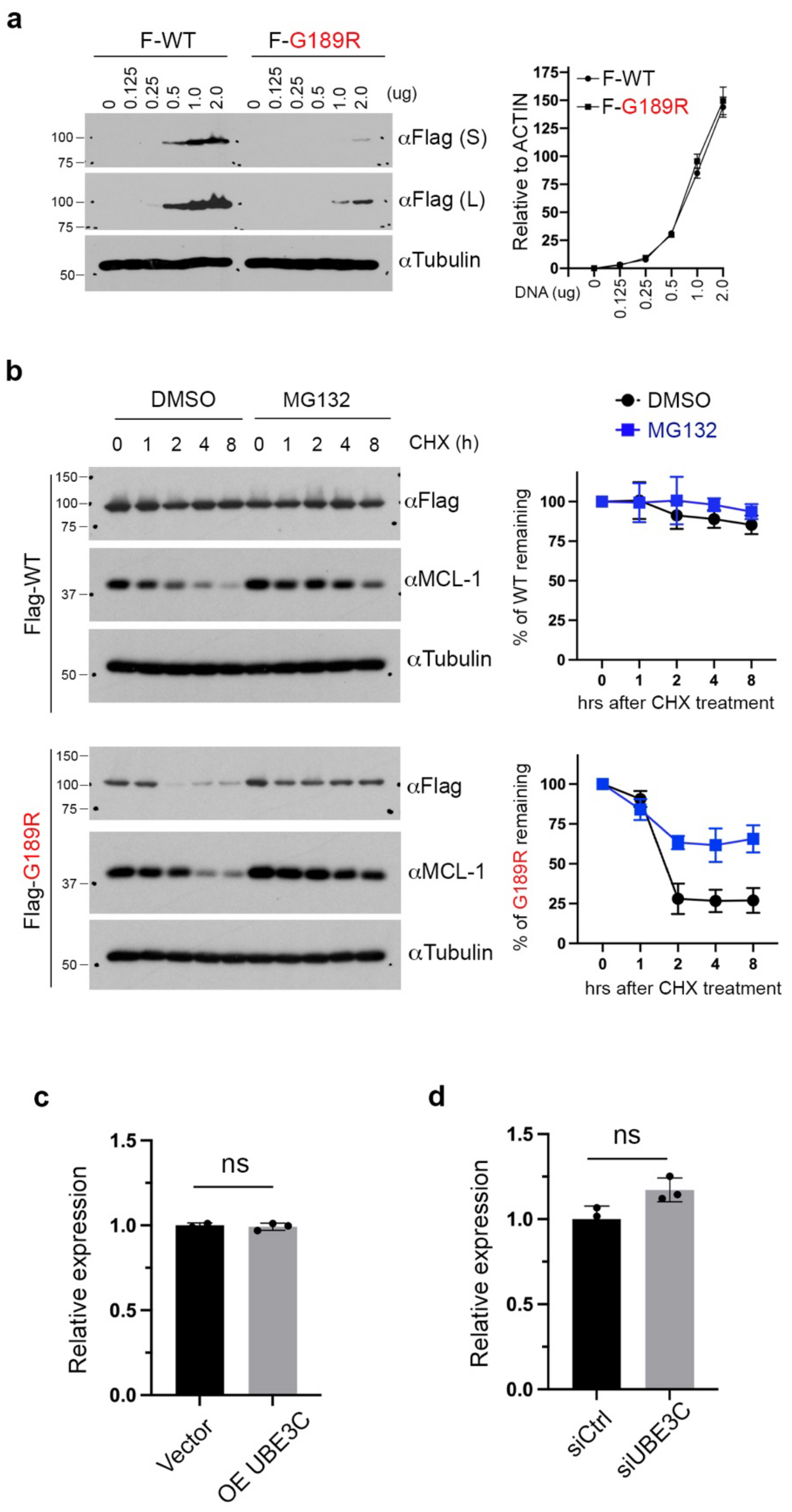

Supplementary Fig. 2: G189R-mutant PRMT9 has a shorter protein half-life than WT PRMT9, and UBE3C does not affect PRMT9 mRNA expression.

- **a.** When cells were transfected with the same amounts of plasmids, G189R-mutant PRMT9 expresses at a much lower level than WT PRMT9 (left panel), whereas their mRNA expression was similar (right panel). n = 3; n is number of independent experiments unless otherwise stated.
- **b.** G189R-mutant PRMT9 has a shorter protein half-life than WT PRMT9 and it is stabilized by the inhibition of proteasome. The stability of WT and G189R-mutant PRMT9 was determined at the indicated time periods after cycloheximide (CHX) treatment in the presence and absence of the proteasome inhibitor MG132 (10 uM). A short-lived protein MCL-1 was used as a control (left panel). PRMT9 western blot signal was quantified using ImageJ software. Error bars represent standard deviation calculated from three independent western blots (right panel). n = 3.
- **c.** Overexpression of UBE3C does not affect PRMT9 mRNA expression. HeLa cells were transfected with either vector control or Flag-tagged UBE3C (OE UBE3C) for 48 h. The levels of PRMT9 mRNA expression were detected using RT-qPCR (n = 3).
- **d.** Knockdown UBE3C does not affect PRMT9 mRNA expression. HeLa cells were transfected with either control siRNA (siCtrl) or siRNA specifically targeting UBE3C (siUBE3C) for 72 h. The levels of PRMT9 mRNA expression were detected using RT-qPCR. ns, not significant. Source data are provided as a Source Data file (n = 3).

Supplementary Fig. 3

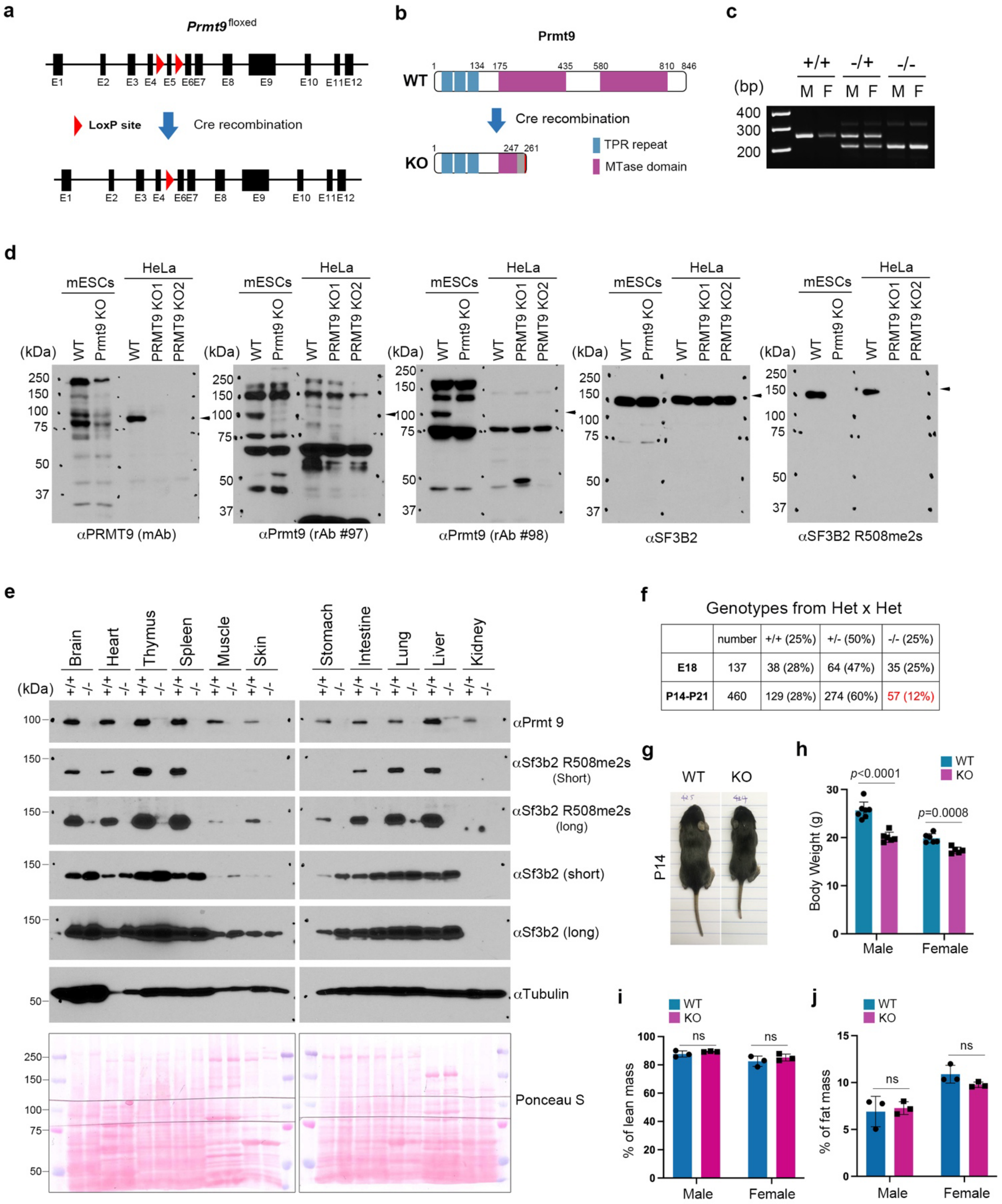

Genotypes from Het x Het

	number	+/+ (25%)	+/- (50%)	-/- (25%)
E18	137	38 (28%)	64 (47%)	35 (25%)
P14-P21	460	129 (28%)	274 (60%)	57 (12%)

Supplementary Fig. 3: Characterization of *Prmt9* knockout mouse model.

- **a.** An illustration of *Prmt9* conditional knockout (cKO) strategy. Two *loxP* sites were inserted into the introns flanking the exon 5 of the mouse *Prmt9* gene.
- **b.** A diagram demonstration of *Prmt9* domain structure. The predicted protein truncation upon exon 5 removal is shown. No functional PRMT9 protein is expected after exon 5 removal.
- **c.** Representative genotyping results of *Prmt9* WT (+/+), heterozygous (+/-), and knockout (KO) (-/-) mice. For *Prmt9* whole-body KO, we crossed *Prmt9*$^{flox/flox}$ conditional mice with a CMV-Cre mouse line that expresses the Cre recombinase in all tissues. CMV-Cre was subsequently removed by crossing with Cre negative mice.
- **d.** Characterization of the newly developed PRMT9 polyclonal antibodies on human and mouse cell lysates. Total cell lysates from WT and *Prmt9* KO mESCs and from WT and *PRMT9* KO HeLa cells were detected by western blot using the indicated antibodies. PRMT9 mAb (clone 128-29-1) detects human *PRMT9*, whereas Prmt9 rAb (#97 & #98) detects mouse *Prmt9*.
- **e.** Western blot detection of PRMT9 and SF3B2 R508me2s in various tissue samples from WT (+/+) and *Prmt9* KO (-/-) mice. Both αTubulin and Ponceau staining were used as loading controls (n = 1).
- **f.** *Prmt9* KO causes partial postnatal lethality. The numbers of *Prmt9* WT (+/+), heterozygous (+/-), and KO (-/-) mice are presented for the indicated stages. E18 refers to embryonic day 18.
- **g.** *Prmt9* KO leads to a smaller body size. A representative image of P14 male mice is shown.
- **h.** *Prmt9* KO leads to a reduced body weight at all developmental stages. Body weight from 8-week-old male and female mice is shown (6M6F, n = 1).
- **i.** and **j.** Body composition analysis of 8-week-old male and female mice, demonstrating that there are no significant differences in terms of lean and fat mass between WT and *Prmt9* KO mice, even though *Prmt9* KO mice are smaller in size (3M3F in each genotype, n = 1). Source data are provided as a Source Data file.

Supplementary Fig. 4

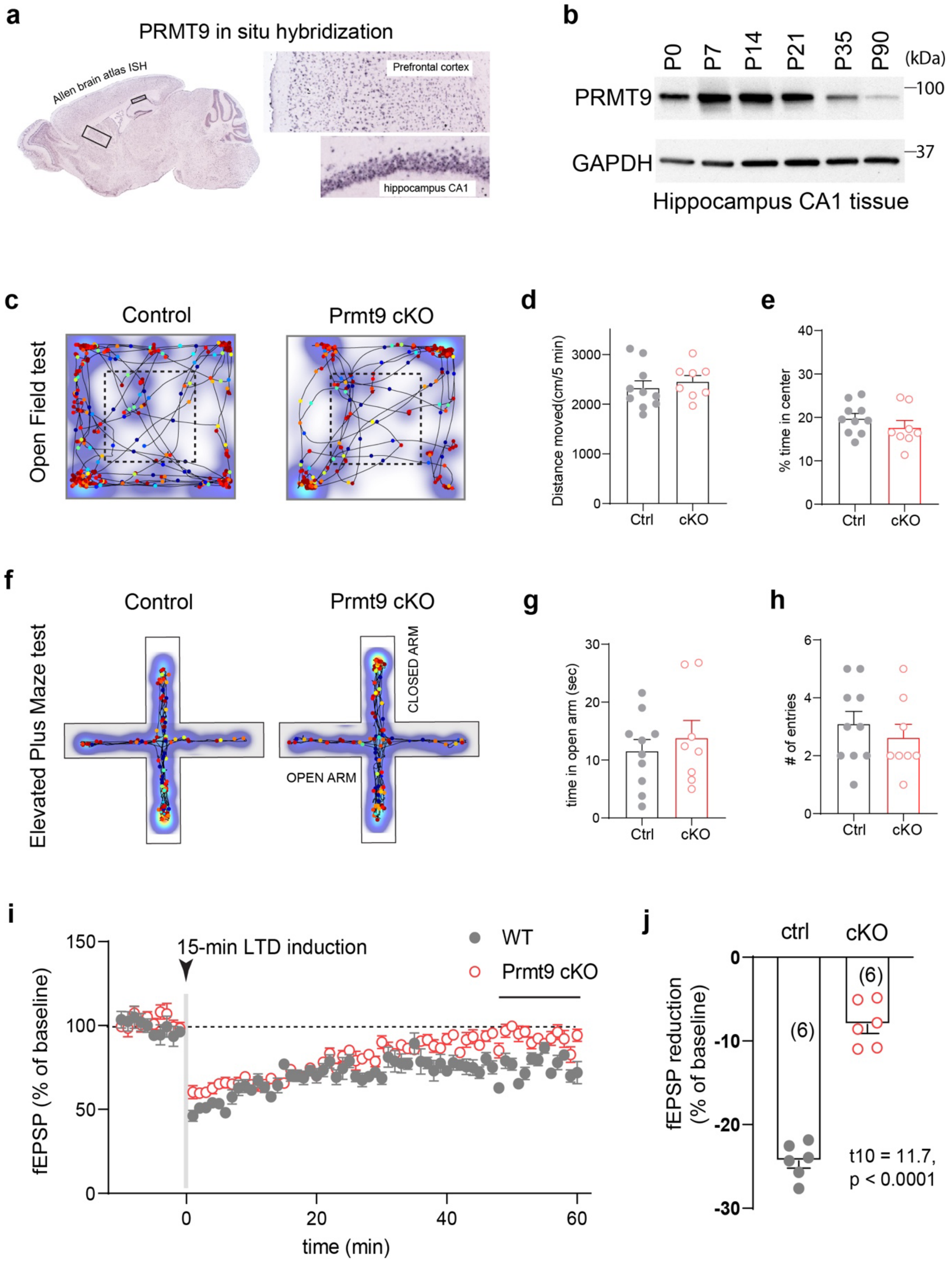

Supplementary Fig. 4: *Prmt9* cKO mice shows normal activity and anxiety related measures.

- **a.** PRMT9 mRNA *in situ* hybridization from Allen Brain Atlas revealed broad levels of expression across cortical (e.g., prefrontal cortex and hippocampus) and subcortical structures.
- **b.** Age-dependent PRMT9 expression indicates that the protein is tightly regulated in early postnatal mouse hippocampus (n = 2).
- **c.** Representative mouse moving tracks/heatmaps in an open field (OF) test.
- **d.** Quantification of OF results shows no difference on the total distance traveled during the 5 min test period.
- **e.** Similar time spent in the center of test area of OF between *Prmt9* cKO and littermate controls.
- **f.** Representative mouse moving tracks/heatmaps in an elevated plus maze (EPM) test.
- **g.** *Prmt9* cKO mice do not differ from littermate controls in the time spent in open arm.
- **h.** *Prmt9 cKO* mice had similar numbers of entry into open arm as littermate controls.
- **i.** *Prmt9 cKO* mice show overall impaired long-term depression (LTD) at the CA3>CA1 synapses.
- **j.** Plot of the last 10 min fEPSP response show *Prmt9 cKO* mice had reduced LTD amplitude (****p < 0.0001). Source data are provided as a Source Data file.

Supplementary Fig. 5

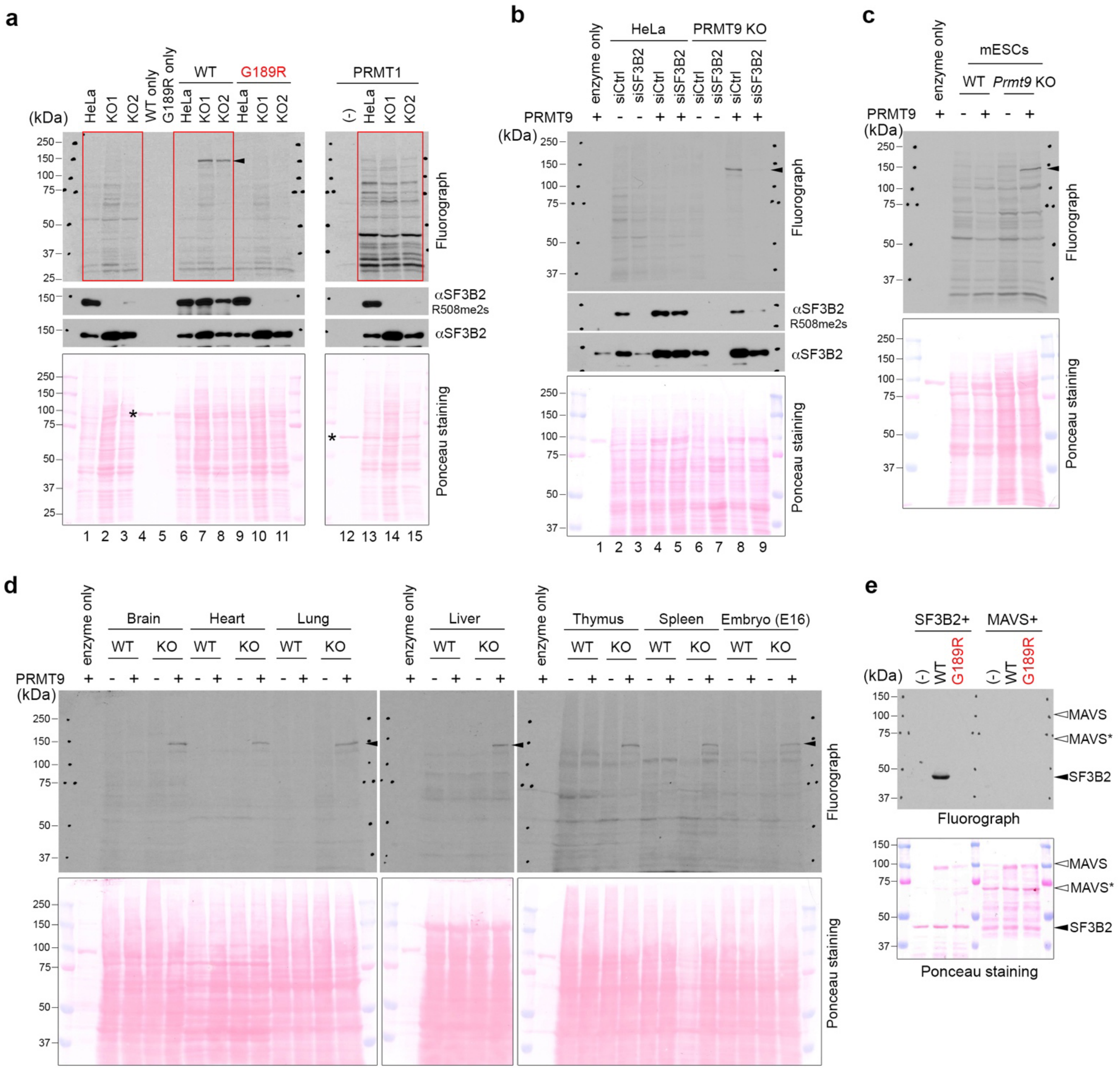

Supplementary Fig. 5: SF3B2 R508 is the primary methylation substrate of PRMT9, and it is highly methylated in cells expressing WT PRMT9.

- **a.** SF3B2 is highly methylated in HeLa cells. *In vitro* methylation assays were performed using ^{3}H-labeled S-adenosylmethionine, recombinant WT and G189R-mutant PRMT9, and the total cell lysates from WT and two different PRMT9 KO HeLa cells (as substrates). PRMT9 can only produce ^{3}H-labeled SF3B2 methylation signal in the KO cells. Methylation of total cell lysates by PRMT1 was set up as a control, demonstrating that PRMT1 methylates a multitude of cellular proteins in both WT and *PRMT9* KO cells. The expression level of SF3B2 and its methylation were detected by western blotting using indicated antibodies. Samples loaded on PVDF membrane was visualized by the Ponceau staining. Black triangle indicates the location of methylated SF3B2, and the asterisk marks the purified recombinant enzymes (n = 2).
- **b.** Similar *in vitro* methylation experiments were performed as described in (**A**), except that we included SF3B2 knockdown total cell lysates to confirm that the ^{3}H-labeled fluorograph signal is specifically from SF3B2 (n = 2).
- **c.** SF3B2 is the primary substrate of PRMT9 in mESCs. Similar to (**A**), except that total cell lysates from WT and *Prmt9* KO mESCs were used as the methylation substrates.
- **d.** SF3B2 is the primary substrate of PRMT9 in various mouse tissues. Similar to (**A**), except that total cell lysates from WT and *Prmt9* KO mouse tissues and from E16 embryos were used as the methylation substrates (n = 1).
- **e.** PRMT9 does not methylate MAVS under the same conditions that methylates SF3B2. *In vitro* methylation assays were performed by incubating WT and G189R-mutant recombinant PRMT9 enzymes with two different GST-tagged recombinant protein substrates, SF3B2 (a.a. 400–550) and MAVS, respectively. Solid triangles indicate the location of SF3B2, and the open triangles mark the locations of recombinant MAVS protein. MAVS* indicates a major degradation product from full length GST-MAVS (n = 1). Source data are provided as a Source Data file.

Supplementary Fig. 6

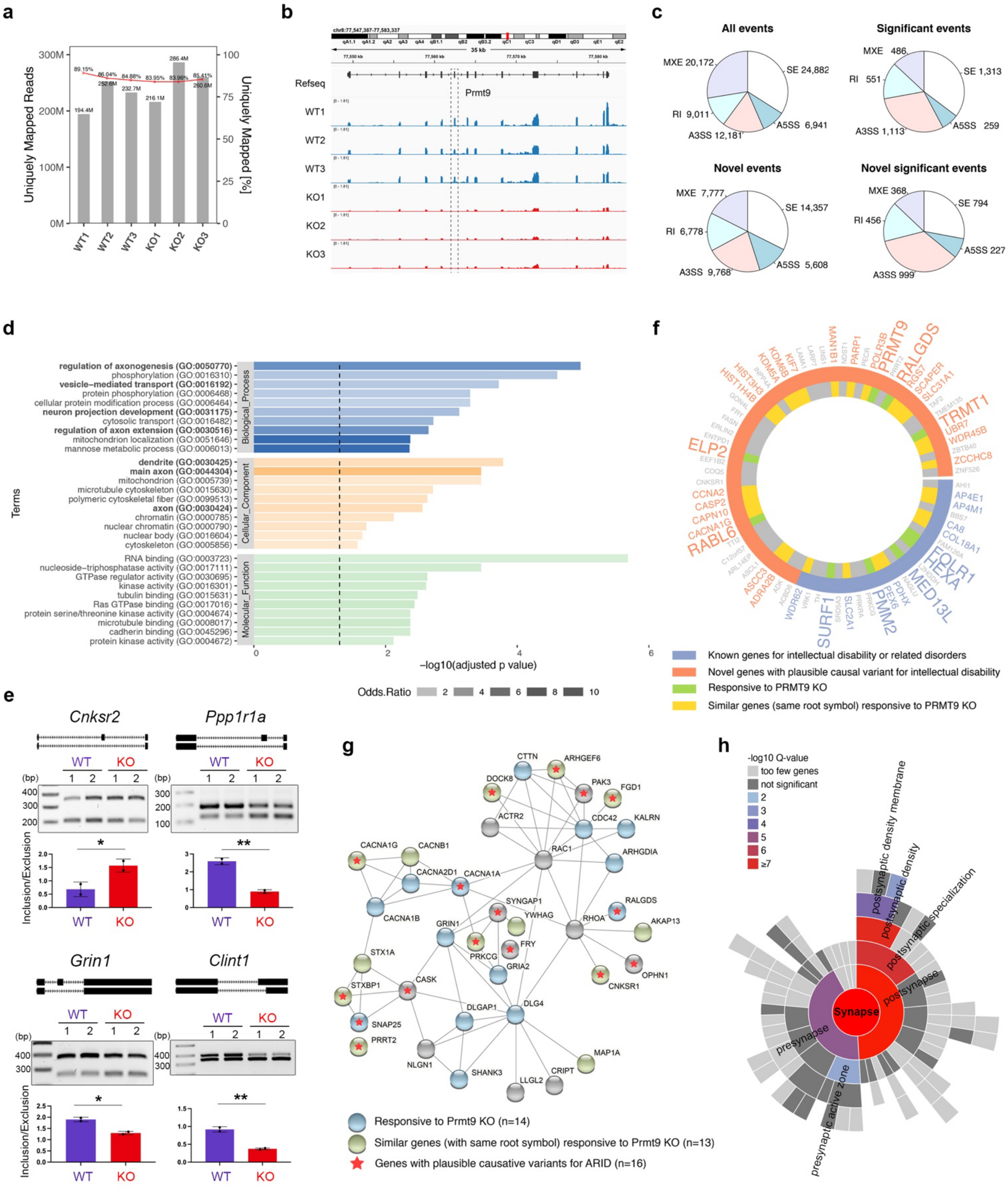

Supplementary Fig. 6: Alternative splicing analysis of RNA-seq data from WT and Prmt9 KO mouse hippocampus.

- **a.** Summary table showing the mapping statistics of the RNA-Seq dataset.
- **b.** Genome browser tracks of the mouse *Prmt9* locus in WT and *Prmt9* KO mice. Exon 5, highlighted by the dashed line is flanked by two *loxP* sites.
- **c.** Pie chart summary of the number of alternative splicing events identified in each indicated category. All events, all of the alternative splicing events identified in the entire RNA-seq dataset; Novel events, alternative splicing events harboring at least one cryptic splice site that is not annotated in the reference genome; significant events, alternative splicing events caused by *Prmt9* KO. SE, exon skipping/skipped exon; A5SS, alternative 5' splice sites; A3SS, alternative 3' splice sites; RI, intron retention/retained intron; MXE, mutually exclusive exon.
- **d.** Gene Ontology (GO) enrichment analysis of alternatively spliced genes (SE and A3SS) upon *Prmt9* KO. The length of bars depicts the Benjamini-Hochberg adjusted p values calculated from a hypergeometric test. Odds ratio of the enrichment were indicated by bar opacity.
- **e.** RT-PCR validation of alternatively spliced genes upon *Prmt9* KO. RNA-seq read coverage across individual alternatively spliced exons in WT and *Prmt9* KO samples is illustrated using Sashimi plots. The number of reads mapped to each splice junction is shown. RT-PCR was performed to validate selected alternative splicing events. Inclusion versus exclusion ratio was calculated. Error bars represent standard deviation calculated from three independent experiments (n = 3).
- **f.** Circular plot demonstration of genes with causative variants associated with autosomal recessive intellectual disabilities (ARID). Genes exhibiting splicing alterations upon *Prmt9* KO are annotated in green, while genes whose paralogs are differentially spliced are annotated in yellow.
- **g.** Prmt9-regualted alternatively spliced genes are enriched in the Ras/Rho/PSD95 interaction network. ARID-related genes are highlighted by a star shape in the center of the nodes. Genes exhibiting splicing alterations upon *Prmt9* KO are annotated in blue. Genes, although themselves are not differentially spliced, but their paralogous genes are, are annotated in green.
- **h.** SynGO enrichment analysis of Prmt9-regulated alternatively spliced events, specifically SE and A3SS. Parental GO terms are shown in inner circles and their corresponding child terms are shown in outer circles. Source data are provided as a Source Data file.

Supplementary Fig. 7

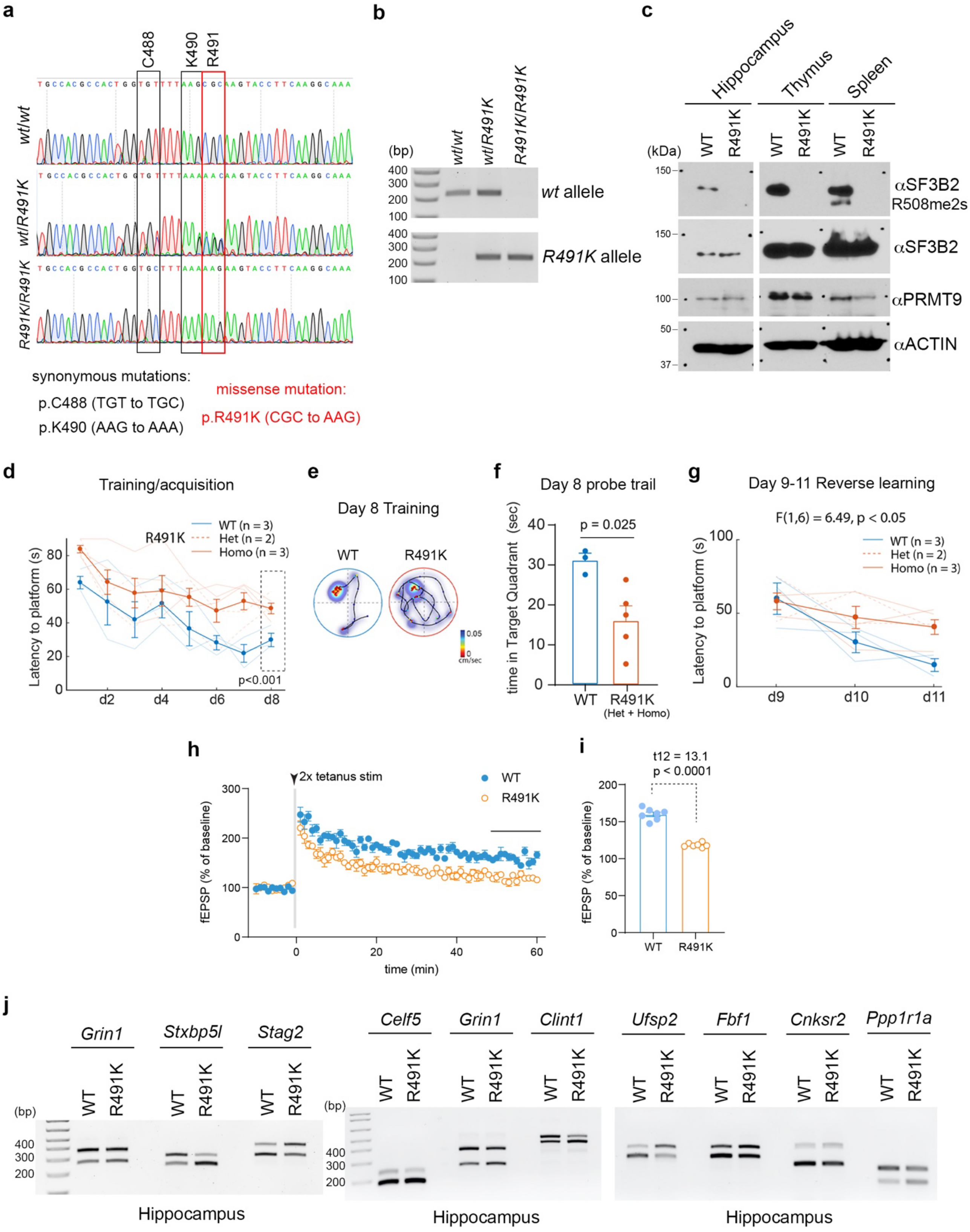

Supplementary Fig. 7: The arginine methylation-deficient mutant Sf3b2 (R491K) mouse model exhibited similar behavior and RNA splicing changes as the *Prmt9* cKO mice.

- **a.** Generation of arginine methylation deficient mutant SF3B2 (R491K) mouse model using CRISPR/Cas9. Mouse Sf3b2 R491 corresponds to human SF3B2 R508. To prevent the re-cutting of the mouse genome by Cas9, two synonymous mutations at C488 and K490 were introduced. The tail DNA was sequenced to confirm the desired mutations.
- **b.** Representative genotyping results of WT, heterozygous knock-in (KI) (*wt/R491K*) and homozygous KI (*R491K/R491K*) mice.
- **c.** Western blot detection of SF3B2 methylation and PRMT9 expression in various tissue samples from WT and R491K homozygous KI mice. Total cell lysates from mouse hippocampus, thymus, and spleen were detected using the indicated antibodies (n = 1).
- **d.** Sf3b2 R491K mice showed slower learning during the acquisition period in the Morris water maze (MWM) experiment. WT, n = 3 (2M1F); Het + Homo, n = 5 (2M, 3F). Y-axis shows the time taken to find the hidden platform during the eight training days.
- **e.** Representative mouse moving tracks and speed on Day 8 of training.
- **f.** Sf3b2 R491K mice showed impaired memory. R491K mice spent significantly less time in the target quadrant during the probe trial (p = 0.025).
- **g.** Sf3b2 R491K mice showed slower learning of the new platform location during Day 9–11 of the reverse learning period. $p < 0.05$ for the effect of genotype.
- **h.** Sf3b2 R491K slices (n = 7) showed reduced level of LTP amplitude during the post-tetanus one hour period.
- **i.** Quantification of amplitude of potentiation using the last 10-min recordings. Sf3b2 R491K (Het and Homo) slices (n = 7) exhibited significantly reduced levels tetanus-induced potentiation of fEPSP slopes.
- **j.** Sf3b2 arginine methylation deficiency (R491K) causes similar splicing changes in mouse hippocampus as *Prmt9* KO. RT-PCR was performed to detect the alternative splicing patterns of PRMT9 target genes using RNA samples from WT and Sf3b2 R491K mutant mouse hippocampus (n = 3). Source data are provided as a Source Data file.

Supplementary Fig. 8

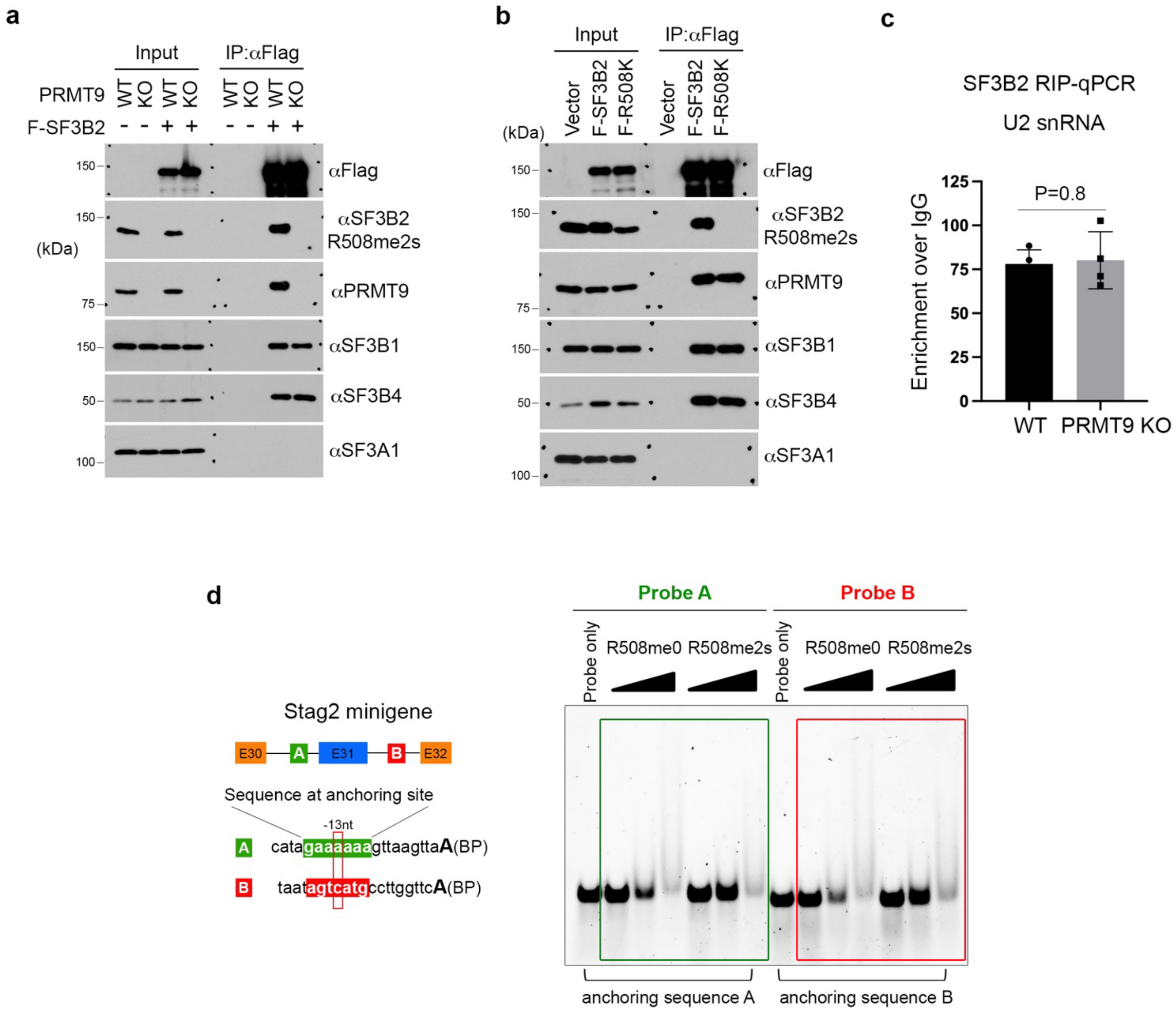

Supplementary Fig. 8: R508 methylation does not affect SF3B2 assembly into the SF3B complex or the interaction of SF3B2 with U2 snRNA.

- **a.** The interactions of SF3B2 with other components of the SF3B complex were not affected in the absence of PRMT9. WT and PRMT9 knockout (KO) HeLa cells were transfected with Flag-tagged SF3B2 (F-SF3B2), and IP-western blot was performed to detect the interactions of SF3B2 with SF3B1, SF3B4, and SF3A1 (n = 2).
- **b.** The arginine methylation-deficient mutation (R508K) does not affect SF3B2 interaction with other components of the SF3B complex. HeLa cells were transfected with Flag-tagged WT and methylation-deficient mutant SF3B2 (R508K). IP-western blot was performed to detect the interactions of SF3B2 with SF3B1, SF3B4, and SF3A1 (n = 2).
- **c.** SF3B2 methylation deficiency does not affect its interaction with U2 snRNA. RNA immunoprecipitation was performed in WT and PRMT9 KO HeLa cells using the SF3B2 antibody. The amount of U2 snRNA in the immunoprecipitated complex was quantified by RT-qPCR. The enrichment was normalized to IgG control (n = 3).
- **d.** SF3B2 R508 methylation (R508me2s) reduces its interaction with anchoring sequence. A diagram illustration of the Stag2 minigene, showing the anchoring sequences of the alternative exon 31 (in green A) and the constitutive exon 32 (in red B). The -13nt is highlighted in a red rectangle. Electrophoretic mobility shift assays (EMSA) were performed to compare the interaction of unmodified (R508me0) and methylated (R508me2s) SF3B2 peptides with upstream (Probe A) and downstream (Probe B) anchoring sequences (n = 3). Source data are provided as a Source Data file.
