# Supplementary material for: Loss-of-function mutation in PRMT9 causes abnormal synapse development by dysregulation of RNA alternative splicing
Source: Nat Commun. 2024 Apr 1;15:2809. doi: 10.1038/s41467-024-47107-9 (PMC10984984; doi:10.1038/s41467-024-47107-9)
Supplement: Supplementary file 3 — Description of Additional Supplementary Information [file 41467_2024_47107_MOESM3_ESM.pdf]

Description of Additional Supplementary Information

File Name: Supplementary Dataset 1

Description: List of proteins identified from vector control, PRMT9 WT, and PRMT9 (G189R) mutation.

File Name: Supplementary Dataset 2

Description: Oligo sequence information for primers and sgRNA.
