# Supplementary material for: Loss-of-function mutation in PRMT9 causes abnormal synapse development by dysregulation of RNA alternative splicing
Source: Nat Commun. 2024 Apr 1;15:2809. doi: 10.1038/s41467-024-47107-9 (PMC10984984; doi:10.1038/s41467-024-47107-9)
Supplement: Supplementary file 6 — Reporting Summary [file 41467_2024_47107_MOESM6_ESM.pdf]

nature portfolio

Corresponding author(s): Yanzhong Yang

Last updated by author(s): Feb 28, 2024

Reporting Summary

Nature Portfolio wishes to improve the reproducibility of the work that we publish. This form provides structure for consistency and transparency in reporting. For further information on Nature Portfolio policies, see our Editorial Policies and the Editorial Policy Checklist.

Statistics

For all statistical analyses, confirm that the following items are present in the figure legend, table legend, main text, or Methods section.

n/a	Confirmed	
☐	☒	The exact sample size (*n*) for each experimental group/condition, given as a discrete number and unit of measurement
☐	☒	A statement on whether measurements were taken from distinct samples or whether the same sample was measured repeatedly
☐	☒	The statistical test(s) used AND whether they are one- or two-sided<br>*Only common tests should be described solely by name; describe more complex techniques in the Methods section.*
☐	☒	A description of all covariates tested
☐	☒	A description of any assumptions or corrections, such as tests of normality and adjustment for multiple comparisons
☐	☒	A full description of the statistical parameters including central tendency (e.g. means) or other basic estimates (e.g. regression coefficient) AND variation (e.g. standard deviation) or associated estimates of uncertainty (e.g. confidence intervals)
☐	☒	For null hypothesis testing, the test statistic (e.g. *F*, *t*, *r*) with confidence intervals, effect sizes, degrees of freedom and *P* value noted<br>*Give P values as exact values whenever suitable.*
☒	☐	For Bayesian analysis, information on the choice of priors and Markov chain Monte Carlo settings
☒	☐	For hierarchical and complex designs, identification of the appropriate level for tests and full reporting of outcomes
☒	☐	Estimates of effect sizes (e.g. Cohen's *d*, Pearson's *r*), indicating how they were calculated

Our web collection on statistics for biologists contains articles on many of the points above.

Software and code

Policy information about availability of computer code

Data collection	pClamp10.2 (Molecular Devices) was used for electrophysiology data collection. Some imaging data were collected using Zeiss Zen software. no commercial, open source and custom code were used in data collection in this study.
Data analysis	ImageJ OS1.52 was used for western blot quantifications. Sequest software was used for mass spectrometry analysis. Bio-Rad CFX Manager 3.1 was used for real time PCR analysis. Imaris software was used to analyze pine head diameter, length, and density. rMATS-turbo (v4.1.0) was used for differential splicing analysis. MaxEntScan software was used for calculating splice site strength. BPP software was used for branch point prediction and comparison. GraphPad Prism 10.0 was used for statistical analysis.

For manuscripts utilizing custom algorithms or software that are central to the research but not yet described in published literature, software must be made available to editors and reviewers. We strongly encourage code deposition in a community repository (e.g. GitHub). See the Nature Portfolio guidelines for submitting code & software for further information.

Data

Policy information about availability of data

All manuscripts must include a data availability statement. This statement should provide the following information, where applicable:

- Accession codes, unique identifiers, or web links for publicly available datasets
- A description of any restrictions on data availability
- For clinical datasets or third party data, please ensure that the statement adheres to our policy

Deep-sequencing data can be accessed with GEO accession number GSE212192. Proteomic data can be accessed from the ProteomeXchange Consortium via the MassIVE partner repository with the dataset identifier PXD050058.

Research involving human participants, their data, or biological material

Policy information about studies with human participants or human data. See also policy information about sex, gender (identity/presentation), and sexual orientation and race, ethnicity and racism.

Reporting on sex and gender	N/A
Reporting on race, ethnicity, or other socially relevant groupings	N/A
Population characteristics	N/A
Recruitment	N/A
Ethics oversight	N/A

Note that full information on the approval of the study protocol must also be provided in the manuscript.

Field-specific reporting

Please select the one below that is the best fit for your research. If you are not sure, read the appropriate sections before making your selection.

☒ Life sciences ☐ Behavioural & social sciences ☐ Ecological, evolutionary & environmental sciences

For a reference copy of the document with all sections, see nature.com/documents/nr-reporting-summary-flat.pdf

Life sciences study design

All studies must disclose on these points even when the disclosure is negative.

Sample size	Sample sizes and number of independent experiments were calculated by power analyses, which takes the pre-specified effect size, experimental variability, and type I and II errors as input arguments.
Data exclusions	For experiments with mice, incomplete data collection and data that are potentially affected by technical errors were excluded from the analysis. No data were excluded from analysis in all other experiments.
Replication	For all experiments, unless stated otherwise, representative analyses from a minimum of three independent experiments are shown. All in vitro analyses using purified recombinant proteins were performed at least three times using 2 different sets of purified proteins. All experiments were successfully replicated. Key conclusions were also validated by different assays. For example, loss of PRMT9 catalytic activity in ID-patient G189R mutation was determined by in vitro methylation assay using recombinant proteins, in vivo methylation detection by western blot, and immunofluorescence. PRMT9 ubiquitination by UBE3C was confirmed by both in vitro and in vivo ubiquitination assays. Changes of alternative splicing caused by PRMT9 loss or hypomethylation of SF3B2 were confirmed by multiple target genes. changes in protein-protein interactions were validated by mass spectrometry and co-immunoprecipitation assays. Multiple human and mouse cell lines and tissues were used to validate that SF3B2 is the primary substrate of PRMT9. Behavioral data scores were independently analyzed by two experimenters.
Randomization	For immunoflurescence staining, fields of view were randomly selected. Randomization was not applicable for all in vitro biochemical assay (in vitro methylation) and cell based assays (western blot, immunoprecipitation, RT-PCR, CLIP-qPCR, electro-physiology), due to the homogeneity of the samples and rigorous controls that are included. In these experiments, group allocation and randomization were unnecessary because all samples were measured independently in the same way in an internally controlled manner. In animal tests, age- and sex matched animals were randomly assigned to different groups using computer generated random tables.
Blinding	Blinding was implemented where possible, in all data collection and analysis process. Groups and animals were coded so that the experimenter is not aware of grouping variables. Blinding was not feasible in the rest of the experiments because knowledge of cell line identity was required to different culture condition.

Reporting for specific materials, systems and methods

We require information from authors about some types of materials, experimental systems and methods used in many studies. Here, indicate whether each material, system or method listed is relevant to your study. If you are not sure if a list item applies to your research, read the appropriate section before selecting a response.

Materials & experimental systems

n/a	Involved in the study
☐	☒ Antibodies
☐	☒ Eukaryotic cell lines
☒	☐ Palaeontology and archaeology
☐	☒ Animals and other organisms
☒	☐ Clinical data
☒	☐ Dual use research of concern
☒	☐ Plants

Methods

n/a	Involved in the study
☒	☐ ChIP-seq
☒	☐ Flow cytometry
☒	☐ MRI-based neuroimaging

Antibodies

Antibodies used

Primary antibodies used in this study were:
Mouse monoclonal anti-FLAG® M2 antibody Sigma-Aldrich Cat# F3165; RRID: AB_259529; 1:2000
Mouse monoclonal anti-β-Actin antibody Sigma-Aldrich Cat# A2228; RRID: AB_476697; 1:5000
Rabbit polyclonal anti-ubiquitin Cell Signaling Technology Cat# 3933; RRID: AB_2180538; 1:1000
Rabbit monoclonal anti-HA-tag antibody (C29F4) Cell Signaling Technology Cat# 3724; RRID: AB_1549585; :1:1000
Rabbit polyclonal anti-GST-tag antibody Cell Signaling Technology Cat# 2622; RRID: AB_331670; 1:2000
Rabbit monoclonal anti-MCL1 antibody (D2W9E) Cell Signaling Technology Cat# 94296; RRID: AB_2722740: 1:1000
Mouse monoclonal anti-α-Tubulin antibody Sigma-Aldrich Cat# T9026; RRID: AB_477593; 1:1000
Rabbit polyclonal anti-SF3B2 antibody Novus Biologicals Cat# NB100-79848; RRID: AB_1110399: 1:2000
Rabbit polyclonal anti-SF3B4 antibody Abcam Cat# ab66659; RRID: AB_2186650; 1:2000
Rabbit polyclonal anti-VCP antibody Bethyl Laboratory Cat# A300-589A; RRID: AB_495512: 1:1000
Rabbit polyclonal anti-UBE3C antibody Bethyl Laboratory Cat# A304-122A; RRID: AB_2621371; 1:1000
Mouse monoclonal anti-SF3B1 antibody (B-3) Santa Cruz Biotechnology Cat# sc-514655;   N/A; 1:500
Rabbit polyclonal anti-SF3A1 Thermo Fisher Cat# PA5-51439; RRID: AB_2647173; 1:1000
Mouse monoclonal anti-PSD95 antibody (K28/43) Millipore Cat# MABN68; RRID: AB_10807979, AB_11212169: 1:100
Rabbit polyclonal anti-Synapsin I antibody Millipore Cat# AB1543; RRID: AB_2200400: 1:100
Mouse monoclonal anti-GluN1 (R1JHL) Millipore Cat# MAB1586; RRID: AB_2279138, AB_11213180; 1:100
anti-GluA1 phosphoSer 845 Millipore Cat# AB5849; AB_92079; 1:100
rabbit polyclonal PRMT9 antibody targeting mouse PRMT9 protein YenZym Antibodies, LLC a mouse PRMT9 peptide (a.a. 794 – a.a. 812), CLDDEVRLDTSGEASHWKQ; 1:1000
Mouse monoclonal PRMT9 antibody, first reported in this publication (PRMT9 is a Type II methyltransferase that methylates the splicing factor SAP145. Nat. Commun. 6:6428. PMCID: PMC4351962); 1:1000
Rabbit polyclonal SF3B2 R508me2s antibody, first reported in this publication (PRMT9 is a Type II methyltransferase that methylates the splicing factor SAP145. Nat. Commun. 6:6428. PMCID: PMC4351962): 1:1000

Validation

All antibodies used are either commercial antibodies that received extensive validation, by producing specific protein bands; or antibodies produces by the authors' groups. For in house made antibodies, we validated with recombinant proteins, and knockout tissues.
anti-FLAG® M2 antibody: https://www.sigmaaldrich.com/US/en/product/sigma/f3165
anti-β-Actin antibody: https://www.sigmaaldrich.com/US/en/product/sigma/a2228
anti-ubiquitin: https://www.cellsignal.com/products/primary-antibodies/ubiquitin-antibody/3933
anti-HA-tag antibody: https://www.cellsignal.com/products/primary-antibodies/ha-tag-c29f4-rabbit-mab/3724
anti-GST-tag antibody: https://www.cellsignal.com/products/primary-antibodies/gst-tag-antibody/2622
anti-MCL1 antibody (D2W9E): https://www.cellsignal.com/products/primary-antibodies/mcl-1-d2w9e-rabbit-mab/94296
anti-α-Tubulin antibody: https://www.sigmaaldrich.com/US/en/product/sigma/t9026
anti-SF3B2 antibody: https://www.novusbio.com/products/sf3b2-antibody_nb100-79848
anti-VCP antibody: https://www.fortislife.com/products/primary-antibodies/rabbit-anti-vcp-antibody/BETHYL-A300-589
anti-UBE3C antibody: https://www.fortislife.com/products/primary-antibodies/rabbit-anti-ube3c-antibody/BETHYL-A304-122
anti-SF3B1 antibody (B-3): https://www.scbt.com/p/sap-155-antibody-b-3
anti-SF3A1 antibody: https://www.thermofisher.com/antibody/product/SF3A1-Antibody-Polyclonal/PA5-51439
anti-PSD95 antibody (K28/43): https://www.emdmillipore.com/US/en/product/Anti-PSD95-Antibody-clone-K28-43,MM_NF-MABN68
anti-Synapsin I antibody: https://www.emdmillipore.com/US/en/product/Anti-Synapsin-I-Antibody,MM_NF-AB1543
anti-GluN1 (R1JHL) antibody: https://www.emdmillipore.com/US/en/product/Anti-NMDAR1-Antibody-all-splice-variants-clone-R1JHL,MM_NF-MAB1586
anti-GluA1 phosphoSer 845 antibody: https://www.emdmillipore.com/US/en/product/Anti-Glutamate-Receptor-1-Antibody-phosphoSer-845,MM_NF-AB5849
rabbit polyclonal PRMT9 antibody targeting mouse PRMT9 protein YenZym Antibodies, LLC a mouse PRMT9 peptide (a.a. 794 – a.a. 812), CLDDEVRLDTSGEASHWKQ
Mouse monoclonal PRMT9 antibody: https://www.emdmillipore.com/US/en/product/Anti-PRMT9-Antibody-clone-128-29-1,MM_NF-

MABE1112
Rabbit polyclonal SF3B2 R508me2s antibody, first reported in this publication (PRMT9 is a Type II methyltransferase that methylates the splicing factor SAP145. Nat. Commun. 6:6428. PMCID: PMC4351962)

Eukaryotic cell lines

Policy information about cell lines and Sex and Gender in Research

Cell line source(s)	HeLa and HEK293 cells were obtained from ATCC.
Authentication	All cell lines used in the project are authenticated and quality assured at the time of receipt, and upon retrieval from stock for studies. Authentication quality control procedures are used as recommended by the American Type Culture Collection (ATCC). This entails (i) Short tandem repeat (STR) DNA fingerprinting for human cell lines and/or (ii) karyotyping to establish identity and rule out cross-contamination.
Mycoplasma contamination	Upon initial receipt of cell lines, whenever cell lines are transferred between laboratories or when cells are brought out for a new set of experiments, we use the Lonza MycoAlert™ Mycoplasma Detection Kit to test all cells for mycoplasma infection. New cell lines are cultivated in a separate quarantine incubator until mycoplasma testing is complete. In rare cases, lines are treated with InvivoGen Plasmocure™ and/or Plasmocin™. Both treatments have shown a good record of success. However, we aim to minimize the instances of any such treatments by ensuring that parental cell lines are mycoplasma free before genetic manipulations are begun. Any contaminated cultures are discarded according to institutional biosafety procedures. Written quality control reports are distributed to the investigators and archived in a shared file system housed in secure "Box" cloud storage.
Commonly misidentified lines (See ICLAC register)	No commonly misidentified cell lines were used.

Animals and other research organisms

Policy information about studies involving animals; ARRIVE guidelines recommended for reporting animal research, and Sex and Gender in Research

Laboratory animals	Mice were used in this study. Emx1-Cre (#005628) and CMV-Cre (#006054) mice (C57BL/6J) were purchased from the Jackson Laboratory. The Prmt9 conditional knockout (cKO) mouse model (C57BL/6) was generated using CRISPR/Cas9 technology (Applied StemCell). The Sf3b2R491K point mutant mouse model (C57BL/6) was generated by CRISPR/Cas-mediated genome engineering (Cyagen). Both sexes were used. Mice age ranges from postnatal day 0 to adult age at ~ 6 months. All data generated using mice as listed in this manuscript are approved by the Institutional Animal Care and Use Committee of the City of Hope (Protocol number 17070) and the University of Arizona (protocol number 13-478).
Wild animals	This study did not involve wild animals.
Reporting on sex	We considered sex as a major biological variable by including both male and female mice in most studies (except bulk tissue RNAseq, which only uses female brain hippocampus tissues). We first analyzed sex-disaggregated data, and found no effects of sex; as such, all data reported in animal studies were pooled from both male and female mice.
Field-collected samples	This study did not involve field-collected samples.
Ethics oversight	All data generated using mice as listed in this manuscript are approved by the Institutional Animal Care and Use Committee of the City of Hope (Protocol number 17070) and the University of Arizona (protocol number 13-478).

Note that full information on the approval of the study protocol must also be provided in the manuscript.

Plants

Seed stocks	This study did not involve seed stocks.
Novel plant genotypes	This study did not involve plants.
Authentication	N/A
